# Ability to predict surgical outcomes by surgical Apgar score: a systematic review

**DOI:** 10.1186/s12893-023-02171-8

**Published:** 2023-09-18

**Authors:** Mina Mirzaiee, Mahdieh Soleimani, Sara Banoueizadeh, Bahareh Mahdood, Maryam Bastami, Amirmohammad Merajikhah

**Affiliations:** 1 Department of Operating Room, School of Paramedical Science, Hamadan University of Medical Sciences, Hamadan, Iran; 2 Bachelor of Surgical Technology, Imam Reza Hospital of Tabriz, East Azerbaijan, Iran; 3 Department of Operating Room, Faculty Member of Paramedical School, Jahrom University of Medical Sciences, Jahrom, Iran; 4 Instructor of Operating Room, Department of Operating Room, School of Allied Sciences, Ilam University of Medical Sciences, Ilam, Iran; 5 Department of Operating Room, Sabzevar University of Medical Sciences, Sabzevar, Iran

**Keywords:** Surgical Apgar score, Postoperative complications, Mortality, Morbidity

## Abstract

**Background:**

The Surgical Apgar score (SAS) is a straightforward and unbiased measure to assess the probability of experiencing complications after surgery. It is calculated upon completion of the surgical procedure and provides valuable predictive information. The SAS evaluates three specific factors during surgery: the estimated amount of blood loss (EBL), the lowest recorded mean arterial pressure (MAP), and the lowest heart rate (LHR) observed. Considering these factors, the SAS offers insights into the probability of encountering postoperative complications.

**Methods:**

Three authors independently searched the Medline, PubMed, Web of Science, Scopus, and Embase databases until June 2022. This search was conducted without any language or timeframe restrictions, and it aimed to cover relevant literature on the subject. The inclusion criteria were the correlation between SAS and any modified/adjusted SAS (m SAS, (Modified SAS). eSAS, M eSAS, and SASA), and complications before, during, and after surgeries. Nevertheless, the study excluded letters to the editor, reviews, and case reports. Additionally, the researchers employed Begg and Egger's regression model to evaluate publication bias.

**Results:**

In this systematic study, a total of 78 studies \were examined. The findings exposed that SAS was effective in anticipating short-term complications and served as factor for a long-term prognostic following multiple surgeries. While the SAS has been validated across various surgical subspecialties, based on the available evidence, the algorithm's modifications may be necessary to enhance its predictive accuracy within each specific subspecialty.

**Conclusions:**

The SAS enables surgeons and anesthesiologists to recognize patients at a higher risk for certain complications or adverse events. By either modifying the SAS (Modified SAS) or combining it with ASA criteria, healthcare professionals can enhance their ability to identify patients who require continuous observation and follow-up as they go through the postoperative period. This approach would improve the accuracy of identifying individuals at risk and ensure appropriate measures to provide necessary care and support.

## Introduction

Surgery has become more accessible for a broader range of diseases and patients due to developments in the field of anesthesia. As a result, more procedures are conducted worldwide each year [1]. It is estimated that 187–200 million cases of surgery are performed worldwide. In addition, it is estimated that one million people die annually within thirty days after surgery [2], and nearly 10% of patients toil from adverse events [3].

Safety and quality are significant matters while providing healthcare services. Since surgery plays a more noticeable role in healthcare globally, safety and quality of such care is receiving growing notice [3].

Reducing perioperative complications/mortality is crucial for patient safety and healthcare economics. It has been observed that approximately fifty percent of post-surgical complications can be prohibited, and advancements in anesthesia-related agents play a significant role in this prevention [4]. Numerous evaluation methods have been suggested to calculation the incidence of post-surgical outcomes/mortality [4]. By objectively assessing these factors, it becomes possible to predict the demand for additional care in intensive care or high-dependency settings and prioritize efforts to reduce surgical complications [5, 6].

Several scoring systems are employed to evaluate surgical patients, such as the American Society of Anesthesiology classification, Revised Cardiac Risk Index, Physiological and Operative Severity Score for the Enumeration of Mortality and Morbidity score, and the National Surgical Quality Improvement Program score. However, these systems of scoring come with limitations specific to them. These limitations include variability in interpretations between different observers, complexities in the calculation, and the need for biochemical investigations [2]. Gawande et al. first presented the SAS implication [7].

A prognostic metric called the SAS is used to forecast post-surgical morbidity/mortality for surgical procedures. This scoring system is straightforward with a range of 0 to 10 points that considers LHR, MAP and EBL during surgery. For first time created for those undergoing vascular and general procedures [8], the SAS has demonstrated its effectiveness in various surgical, containing urological, gynecologic, orthopedic, and neurosurgery [9]. The SAS is a comprehensive tool that provides a detailed assessment of the clinical and biological status of the patient, aiding in predicting mortality [10]. However, studies investigating the effectiveness of SAS have yielded conflicting results. While some studies support its value in predicting postoperative outcomes, others focusing on gastric, neurosurgery, and orthopedic patients have been unable to establish a consistent relationship [11–13]. According to Pittman et al., the SAS exhibits a modest postoperative morbidity discrimination level and mortality across a variety of surgical specialties [14].

Considering that no study has so far comprehensively focused on the effect of the Apgar score on the prediction of surgical results, the current review study investigated SAS results on the prediction of surgical outcomes.

## Materials and methods

### Data collection

The PRISMA checklist and flowchart were used to evaluate the retrieved studies and improve their quality to identify the relationship between SAS and surgical complications [15].

### Search strategy

The relevant literature was thoroughly searched within the PubMed, Medline, Scopus, Web of Science, and Embase databases until June 2022 with no time or language limitation using keywords such as (‘Surgical Apgar score’ AND ‘Complication’ AND ‘Predict’). To conduct the study, three authors initially reviewed the sources of qualified article reports and subsequently evaluated the abstracts and titles of the identified articles. Irrelevant, duplicate, and non-original essays were excluded from further investigation. The relevant data, including the first author, publication year, demographic characteristics of countries, participants, and treatment options for each group, were extracted using a predefined standardized procedure. All these tasks were carried out independently by the authors.

### Inclusion and exclusion criteria

The entry criteria included the correlation between SAS and any modified/adjusted SAS (m SAS,^1^ eSAS,^2^ M eSAS,^3^ and SASA), and complications before, during, and after surgeries. On the other hand, letters to the editor, reviews, and case reports were excluded from the study.

### Data extraction

The process of article selection involved three investigators (MM, MS, and MB), who independently carried out the screening. In case of disagreements, a third author was involved in reaching a consensus. The data extraction process utilized a datasheet that included year of publication, first author, country, study design, SAS, modified or adjusted SAS, complications occurring before, during, and after surgeries, and article quality.

### Quality assessment

The methodological quality of the articles was assessed following the guidelines provided by the Newcastle and Ottawa statements. These guidelines were used as a framework for evaluating the quality of the included studies during the review process [16]. In this guideline, criteria were considered to check the selection of subjects under study, their comparability, exposure, and outcome, and at most nine stars were assigned to each study. Studies with seven or more stars and six stars or less were classified as high and low-quality, respectively. The potential for bias in the study results was examined independently by two researchers. In cases where disagreements arose, the researchers resolved them through discussion and negotiation. This process ensured a comprehensive assessment of potential bias in the study findings.

## Results

**Fig. 1:**
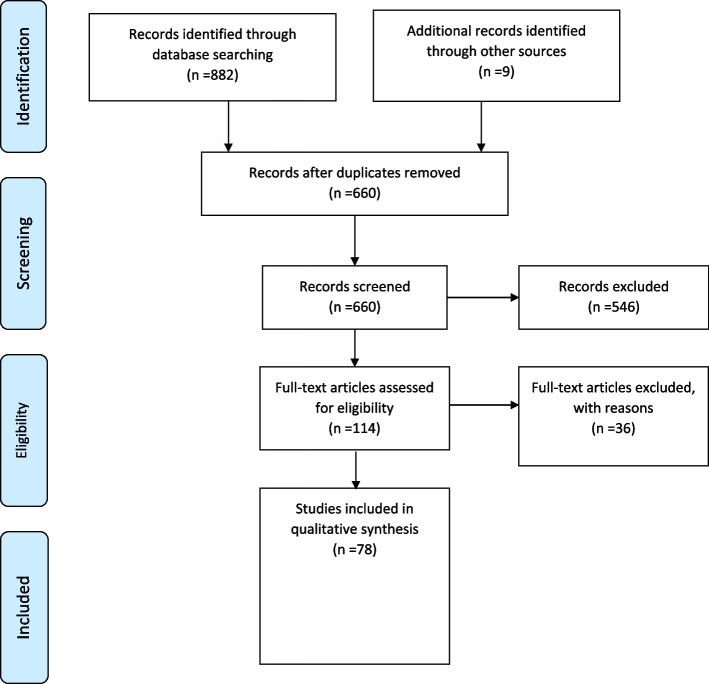
Flow diagram of the study selection for the review process

The SAS is a straightforward scaling model that uses easily computed and recorded. It provides surgeons and anesthesiologists a tool to recognize patients at a higher risk for adverse outcomes or complications. By utilizing the SAS, healthcare professionals can effectively assess the risk profile of patients and make informed decisions regarding their care and management. Therefore, the present study investigated and searched for information about SAS this species. To achieve this goal, PubMed, Scopus, Embase, Web of Science, and Google Scholar databases were searched using keywords ((surgical Apgar score) AND (complication)) AND (predict). In the initial search, 882 articles were found from four databases, the Google Scalar search engine and nine additional records were identified through the other sources. Overall, 660 duplicates were excluded with the help of EndNote X8 software, and 114 articles remained, among which 36 articles were excluded after reviewing their full texts, and 78 articles remained for full-text screening (Fig. 1 The remaining 78 articles included 28, 26, and 22 retrospectives, cohort, and prospective studies, respectively, and two studies were randomized control trials (Tables 1, 2, 3, and 4).

**Table 1:** (General-Vascular- Oncologic- Neuro)Surgery

NO	Author(s)	Year	Type of study	Type of surgery	Number of patients	Article Findings	Surgical Apgar score	Main predicts
1	Scott E. Regenbogen [17]	2010	Cohort study	Colon and rectal resection	795	The SAS was a valid measure to predict post colectomy tolls. Therefore, late complications could be associated with surgery situations	SAS(0–4)	• Surgical site infection • deep venous thrombosis/pulmonary embolism, • Bleeding • Renal failure • Peripheral nerve injury • Myocardial infarction • Stroke • Pneumonia • Unplanned intubation or prolonged • Ventilation
2	Scott E, Regenbogen [18]	2008	Cohort study	General and Vascular surgery	4119	Components of patient susceptibility, procedure complexity, and operative performance are integrated in the Surgical Apgar Score, which provide a measure of immediate postoperative condition and prognostication beyond standard risk—adjustment	SAS < 4(A: major complications) SAS (0–2) B	• A: acute renal failure • Bleeding • cardiac arrest • coma • deep venous thrombosis • myocardial infarction • unplanned intubation • ventilator use for 48 h • pneumonia • pulmonary embolism • stroke • wound disruption • surgical site infection • sepsis • septic shock • systemic inflammatory response syndrome (sirs) • vascular graft failure • B: dying from that complication
3	Keevan singh [19]	2019	Retrospective observational cohort	emergency abdominal surgery	220	The SAS, which is used in those who underwent emergency procedures, helps identify patients at a higher peril of post-surgical outcomes	SAS ≤ 4	• Acute renal failure • Bleeding • cardiac arrest • coma • deep venous thrombosis • myocardial infarction • unplanned intubation • ventilator • pneumonia • pulmonary embolism • stroke • wound disruption • surgical site infection • sepsis • septic shock • systemic inflammatory response syndrome • vascular graft failure, • death
4	Julia B. Sobol [20]	2013	Retrospective cohort study	High-Risk Intraabdominal Surgery	8501	The SAS and clinical decisions are strongly related considering ICU entrance after high–risk Intra-abdominal procedure	SAS (0–2)	• Mortality rate • ICU admission
5	Astushi Sugimoto [21]	2022	Retrospectively	radical surgery OF Colorectal cancer	639	Low Apgar score is an independent predicting characteristic for cancer-specific survival after surgery. SAS may be a valuable biomarker foreseeing oncological results in Colorectal cancer	SAS ≤ 6	• Overall survivor • Cancer specific survival • Recurrence-free survival
6	Yoshito Tomimaru [22]	2018	Retrospective cohort	Hepatectomy For Hepatocellular carcinoma	158	SAS can predict the after-surgical complications following hepatectomy for hepatocellular carcinoma	SAS ≤ 6	• Pleural effusion and/or ascites • Cardiopulmonary • Bile leakage • Ileus • Intra-abdominal abscess • Liver failure Wound infection
7	Y Toyonaga [23]	2017	Retrospective cohort	Emergency abdominal or cerebral surgery	742	A raised danger of post-surgery acute kidney injury (AKI) and mortality was observed in patients with SIRT and SAS scores	SAS < 5	• Post-operative Acute kidney injury • Hospital mortality
8	Julio Urrutia [24]	2015	Prospective study	Major and intermediate spinal surgeries	268	Danger can be stratified using Surgical Apgar Score. It is also able to discriminates patients undergoing spine surgery	SAS < 4	• 30-day major complication • Death
9	Takanobu Yamada [25]	2016	Retrospective cohort	Gastrectomy For cancer	190	SAS is helpful in prognosis survivorship after Gastric procedure	SAS ≤ 6	• Overall survival
10	Wie Yu [26]	2016	Cohort	Gastrointestinal surgery	41	Malignant obstructive jaundice patients with higher preoperative brain natriuretic peptide(BNP) level and lower SAS were recognized at high danger of major adverse cardiac events(MACE) following surgery	SAS < 4	• Heart failure • cardiac insufficiency • cardiac asthma • severe arrhythmia • myocardial infarction
11	John E. Ziewacz [27]	2013	Retrospective cohort study	Neurosurgery	918	The surgical Apgar score predicted postoperative mortality up to 30 – day, the rate of complication, and extended ICU and hospital stay	SAS(0–2)	• Death • coma of more than 24 h duration • acute renal failure • Postoperative bleeding • requiring ICU stay • unplanned intubation • ventilation • pneumonia • cardiac arrest • myocardial infarction • pulmonary embolism • infection • sepsis • Systemic inflammatory response syndrome • Pseudomeningocele formation • deep vein thrombosis • cerebrovascular accident
12	Israel Zighelbiom [28]	2010	Cohort study	Cytoreductive surgery	267	SAS can strongly predict post-surgery outcomes in those experiencing cytoreductive procedures for advanced epithelial ovarian cancer	SAS ≤ 4	• Readmission < 30 days • ICU admission • Venous thromboembolism, • Blood transfusion ≥ 4 U red blood cell • Wound disruption • Acute renal failure • Pneumonia • Postoperative ventilator support ≥ 48 h • Sepsis • Inflammatory Response Syndrome • Uplanned intubation • Need for reoperation • Estimated blood loss ≥ 2000 mL • Acute myocardial infarction
13	Monika Zdenka Jering [29]	2015	Retrospective study	General, vascular, or general oncology surgery	4,728	SAS can predict the risk of main post-surgery outcomes in the patient within 30 days after general, vascular, or general oncology procedure	SAS (0–4)	• ventilator use for more than 48 h • wound disruption • deep or organ space surgical site infection • renal failure sepsis
14	Jakub Kenig [30]	2018	Prospective study	Emergency abdominal surgeries	315	It is confirmed that the SAS confirmed is a straightforward and strong prophesier of 30-day post-surgery morbidity/mortality in those who underwent emergency abdominal procedures	SAS(0–4)	• major postoperative complications • death
15	Jakub Kenig [31]	2018	Prospective study	Abdominal cancer surgery	164	SAS are undeniably prognoses of 30-day post-surgical outcomes in elders who underwent elective abdominal cancer procedures	SAS < 7	• 1—year mortality • postoperative outcomes
16	Marco La Torre [32]	2013	Retrospective study	pancreatic surgery	143	The SAS is utilized to recognize those who at danger of main outcomes and dying after pancreatic procedures and optimize the use of hospitalization	Not applicable SAS ≤ 5 (B)	• Mortality rate • surgical site infections • biliary fistulas • B: pancreatic fistula
17	Antonio Masi [33]	2017	Retrospective study	major/extensive intra-abdominal surgery	629	Based on the SAS, veterans are at high danger for poor postoperative consequence major/extensive intra-abdominal surgery	SAS ≤ 4 A SAS(5–6) B	• A: (Failure to wean from ventilator • acute renal failure • return to the operating room • sepsis • 30, 60, 90-day mortality rates) • B: overall morbidity
18	Toru Aoyama [34]	2016	Retrospective study	pancreatic surgery	189	Considerable risk factors for surgical tolls after pancreatic surgery included the SAS and body mass index	SAS (0–4)	• Delayed gastric emptying • pancreatic fistula • abdominal abscess • surgical site infection • postoperative bleeding
19	Muhammad Z. Arifin [35]	2021	Prospective study	Traumatic brain injury	123	The SAS has a correlation with outcomes in thirty days post-surgery in patients with brain procedures	Not applicable	• Wound dehiscence • acute kidney injury • pneumonia, Seizures • sepsis or shock septic • cardiac arrest • re – intubation/ventilator • re – operation • Neurological deficit • Coma • transfusion > 4 units
20	M. Mura Assifi [36]	2012	Retrospective study	Pancreaticoduodenectomy	553	This score is a prominent predictor of perioperative complications for those who underwent Pancreaticoduodenectomy	SAS ≤ 4 (Group A) SAS = 4 (B)	• Group A; Delayed gastric emptying, • Intra-abdominal abscess requiring drainage • Cardiac arrhythmia • Pulmonary complications • B = pancreatic fistula • SAS was not a predictor for mortality
21	Iulian Buzincu [37]	2021	Prospective observational study	Oncologic surgery	205	SAS can beneficially detect cancer procedure patients at threat for post-surgical cardiovascular and metabolic dysfunction. SAS had a low distinction ability to detect between those with the probability of developing postoperative complications and those without it	SAS = 7	• Cardiovascular dysfunction • renal dysfunction • organ dysfunction • mortality rate • metabolic dysfunction
22	Mirjana Cihoric [38]	2016	cohort study	Emergency high-risk abdominal surgery	355	The SAS can significantly predict, yet weakly discriminate between main outcomes and mortality among those who underwent emergency abdominal procedures	SAS (0—2)	• Post-surgical abdominal wall dehiscence • surgical site bleeding • upper gastrointestinal bleeding • ileus • wound infection • intra-abdominal infection/abscess • anastomotic leakage, • Death • ICU admission
23	Kyle S. Ettinger [39]	2016	Retrospective cohort study	Microvascular head and neck reconstruction	154	SAS is not probably a powerful score for danger stratification in who underwent major head and neck reconstruction with fibular flaps	Not applicable	• Can’t predict
24	Neha Goel [40]	2018	Prospective study	Elective major cancer surgery	405	The SAS was not widely capable to accurately predict danger serious complication of postoperative at the patient level	SAS = 0–4	• Returned to the operating room • urinary tract infection • respiratory complication • wound complication • cardiac complication
25	Sudarshan Gothwal [41]	2018	Analytical observational study	Abdominal Surgery	Group (A) = 25 Group (B) = 25	SAS is a beneficial metric to distinguish the patient undergoing laparotomy complications	Mean SAS in group A = 4.9 the mean SAS in group B = 7.88	• ARF • faecal fistula • intraabdominal abscess • mortality • Pneumonia • prolonged ventilation • wound dehiscence • Main outcomes or death within 30-days
26	Shih-Yuan Hsu [42]	2017	Retrospective study	Intracranial meningioma surgery	99	SAS can absolutely predict the main outcomes of those who underwent cranial procedures	SAS (0 – 3)	• Deep venous thrombosis • Pneumonia Stroke • Wound disruption • Deep or organ-space surgical site infection • Sepsis • Systemic inflammatory response syndrome • death
27	Mitsiev, I [43]	2021	Retrospective study	Hepatectomy	119	SAS can predict risk for major postsurgical complications following hepatectomy, and might be helpful in improving the overall patient outcome	SAS (3–4)	• biliary leak • bleeding • hematoma • wound dehiscence • Died • Pleural effusion • atelectasis
28	Kousei Miura [44]	2022	retrospective case–control study	Cervical Spine surgery	261	Considerable risk factors for main outcomes after cervical spine procedure included lower SAS, higher Controlling Nutritional Status Score, and longer operative time	Not applicable	• Pneumonia • Unplanned intubation • Bleeding • Sepsis • severe delirium • venous thrombosis • stroke • pulmonary embolism • wound disruption
29	Gajanthan Muthuvel [45]	2014	Retrospective study	Emergency general surgery	3,968	SAS and length of stay (LOS) and Anesthesiologists Physical Status Classification (ASA) class could intensely predict readmission following emergency general surgery	SAS < 6	• Readmission rate
30	Christian Ngarambe [46]	2017	Retrospective study	laparotomy	218	SAS could well predict postoperative mortality statistic and main complication after laparotomy	SAS(0–4)	• Deaths • Deep wound infection • Reoperation
31	Ohlsson, H [47]	2011	Retrospective study	General & Vascular surgery	224	Strong relationship between SAS with main outcomes within 30 days after General & Vascular procedures	SAS(0–4)	• Acute renal failure • Bleeding, Cardiac arrest • Coma • Deep venous thrombosis • Septic shock • Myocardial infarction • Unplanned intubation • Ventilator use 48 h • Pneumonia • Pulmonary embolism • Stroke • Wound disruption • Deep or organ space surgical site infection • Sepsis • Systemic inflammatory response syndrome • Vascular graft failure • Death
32	Chien-Yu Ou [48]	2017	Retrospective study	Lumbar fusion surgery	199	SAS was a predictor score for significant outcomes in spinal procedures	SAS(0–2)	• red cell transfusions > 4 Unit • pneumonia • deep surgical site infection • systemic inflammatory response syndrome
33	K.E. Padilla-Leal [49]	2021	Prospective observational study	Gastrointestinal oncologic surgery	50	SAS was a predictive characteristic of post-surgical at 30 days in gastrointestinal surgery	SAS(0–4)	• Infectious • abdominal sepsis • surgical wound infection • urinary tract infection
34	Sílvia Pinho [50]	2018	cross-sectional prospective observational study	colorectal surgery	358	SAS was related to making accurate clinical decisions for admissions to the intensive care units after colorectal procedures	Not applicable	• Cardiovascular rhythm disorders • Cardiac arrest • Respiratory hypoxia • Subcutaneous emphysema • Pulmonary aspiration • Bronchospasm • Bleeding
35	Atul A Gawande [51]	2007	Retrospective study	General & Vascular surgery	303	SAS usefully rate the patients’ condition following general or vascular operation	SAS(0–4)	• acute renal failure • bleeding • cardiac arrest • coma • deep venous thrombosis • septic shock • MI • unplanned intubation • ventilator use for 48 h or longer • pneumonia • pulmonary embolism • stroke • wound disruption • surgical site infection • sepsis • systemic inflammatory response syndrome • vascular graft failure
36	Marcovalerio Melis [52]	2017	prospectively	General Surgery	2153	Veterans at high risk for postoperative tolls can be effectively identified by the SAS	SAS < 5	• Overall morbidity • 30-day mortality

**Table 2:** (Orthopedic-Urologic-Gynecologic-Thoracic) Surgery

NO	Author(s)	Year	Type of study	Type of surgery	Number of patients	Article Findings	Surgical Apgar score	Main outcome
1	Sanja Sakan [53]	2015	Cohort study	Hip fracture surgery	43	The SAS was a noteworthy indicator for estimate the 30—days major surgical outcomes real feedback data about post-surgery danger can be provided in the operating theatre using the SAS	SAS ≤ 4 (A)	• A: ICU length of stay • postoperative bleeding • cardiac arrest • myocardial infarction • deep venous thrombosis • pulmonary embolism • stroke • unplanned intubation • mechanical ventilation • pneumonia • Sepsis • septic shock • acute renal failure • B:SAS wasn’t able to predict 30 days and 6month mortality
2	Christian Wied [54]	2016	Retrospective observational cohort study	Trans tibial amputation or trans femoral amputation	170	SAS is directly associated considering the development of complications following Trans femoral amputation. this score is specifically helpful when patients are split into high and low-risk classes SAS model doesn’t have any predictive importance in the trans tibial amputation	SAS < 7(Trans femoral)	• Death • Bleeding • Sepsis • Acute myocardial infarction/acute heart failure • Acute renal failure • Pneumonia • Stroke • Pulmonary embolism
3	Thomas H. Wuerz [55]	2011	Retrospective cohort	Hip and knee arthroplasty	3236	SAS couldn’t provide any sufficient data about complication of surgery in patients	Not applicable	• Can’t predict
4	William D. Stoll [56]	2016	Retrospective longitudinal cohort	Kidney transplant	204	Patient and surgical risk can be assessed by SAS through providing utility within kidney transplantation	SAS ≤ 7	• Risk of ICU admission • Cost of hospitalization • Hospital Readmission • A history of stroke • ICU admission following transplant • high hospital costs
5	Atsushi Kotera [57]	2018	Retrospective study	Femoral neck surgeries	506	This score is a helpful device for assessing post-surgical outcomes in people who have undergone a femoral neck procedure SAS in combination with ASA 3 or with significant risk factors was remarkably able to estimate post-surgical outcomes	SAS ≤ 6	• Pneumonia • Venous thrombus • Surgical site infection • Postoperative heart failure • Sepsis, Stroke • Bleeding • Acute myocardial infarction
6	Timothy Ito [58]	2015	Prospective study	Radical or partial nephrectomy	886	The SAS can recognize patients at higher danger for main outcomes and dying after renal lump incision	SAS ≤ 4	• hemorrhage • cardiac events • pulmonary events • pneumonia • unplanned intubation • Stroke • wound disruption
7	MatthiasOrberger [59]	2017	Retrospective study	Radical prostatectomy	994	SAS was not associated with Negative outcomes of robot-assisted laparoscopic transperitoneal radical prostatectomy	SAS = 7	• Cardiopulmonal • Thrombembolic • Surgical site Infection • Bleeding • Prolonged Catheterization
8	Farhan Haroon [60]	2021	Prospective observational study	Hip fractures	150	SAS showed trust feedback data about the patient’s postoperative danger during the hip fractures surgery	SAS ≤ 4	• Pulmonary and cardiac complications • Unable to predict kidney complications
9	Masato Hayashi [61]	2019	Retrospective observational study	Trans thoracic esophagectomy	190	SAS can predict postoperative tolls transthoracic esophagectomy surgery	SAS <	• Anastomotic leakage • respiratory and cardiac complication • nerve palsy • chylothorax
10	Akihiro Nagoya [62]	2022	Retrospective study	Lung resection	585	This score was an insignificant risk factor for lung cancer	SAS < 7 A: (short-term outcomes) SAS < 7 B: (long-term outcomes)	• A: Cardiopulmonary • Myocardial infarction • Prolonged air leak • Pneumonia • Nerve palsy • Postoperative bleeding • Empyema • Chylothorax • Atelectasis • Airway stenosis • Respiratory failure • ARDS) َAdult Respiratory Distress Syndrome) • Bronchial fistula • Pulmonary edema • Pleural effusion • Surgical site infection • Delirium • Stroke • Gastrointestinal • B: disease-free survival, overall survival rate

11	Kojiro Eto [63]	2016	Prospective study	Esophagectomy	399	The SAS is taken into account as beneficial in predicting the post-surgical morbidities development after esophagectomy for esophageal cancer	SAS < 5	• Pulmonary complication • cardiovascular morbidities • anastomotic leakage • anastomotic strictures • surgical site infection • morbidity
12	Danica N. Giugliano [64]	2017	Prospective study	Esophagectomy	212	This score is a considerable predictor of outcomes and hospitalization time for patients who underwent esophagectomy	SAS (1–2 or 3–4) (Group A) SAS ≤ 2 (B) SAS (0–2) (C)	• (Group A) = Arrhythmia, respiratory complications, Pneumonia, sepsis, UTI, Chylothorax • B = anastomotic leak • C = length of stay in hospital
13	Makoto Yamamoto [65]	2021	Retrospective cohort study	Gynecological cancer surgery	173	ability to predict post-surgical outcomes and mortality among 1 year	SAS ≤ 6	• Post-operative major complication • Death within1year • Post-operative intensive care
14	Kazumi Kurata [66]	2017	Retrospective study	Gynecological Surgeries (non-laparoscopic surgeries)	68	The indication of solemn dangerous outcomes in geriatric gynecological within 30 days	AS ≤ 6	• Gastrointestinal anastomotic failure • ureteral fistula • hemorrhagic shock • circulatory failure • heart failure • pleural effusion

15	Geetu Bhandoria [67]	2020	Prospective observational study	Gynecologic oncological surgeries	100	Prediction complications who those underwent oncological procedures	SAS ≤ 5	• Cardiac dysfunction • Neurological dysfunction • Gastrointestinal and renal dysfunction
16	Geetu Bhandoria [68]	2017	Rct	Surgery for gynecological malignancies	100	Low SAS prominent is associated with morbidity in women experiencing gynecological malignancies surgery	SAS ≤ 5	• DVT • Incomplete wound dehiscence • Post operation ventilator support Reoperation
17	Rachel M. Clark [69]	2015	Retrospective cohort study	Hysterectomy surgery	632	Low Surgical Apgar Score unable to estimate which patients will have postoperative tolls	SAS ≤ 4	• Re operation • Fistula • Anastomotic leak • Pulmonary embolism • Bowel obstruction • Urinary injury • Nerve Injury • Vascular injury • Unplanned ICU admission • Death • Hospital stay • Readmission
18	Nakagawa, A [70]	2017	Retrospective study	Esophagectomy	400	SAS was beneficial in predicting short and long term complications after esophagectomy	SAS ≤ 5	• Mortality • Pneumonia • gastric conduit necrosis • gastrointestinal anastomotic leak • bronchial fistula • acute ischemic heart disease • subarachnoid hemorrhage • lower survival rate
	Julio Urrutia [13]	2012	Prospective study	general orthopaedic surgery	723	30-day main outcomes after orthopedic procedure were not predicted by SAS	Not applicable	• Can’t predict

20	Sandip M. Prasad [71]	2009	Retrospective study	Radical Cystectomy	155	Death in those who underwent radical cystectomy can be predicted by the SAS	Not applicable	• Not applicable
21	Amy C. S. Pearson [72]	2017	Retrospective Study	Liver Transplantation	628	This score predicted morbidity and mortality after liver transplant	Not applicable	• Death or any severe complication • Sepsis • Reintubation • New dialysis • Seizure • Myocardial infarction • Stroke • Pulmonary embolus • Death • Postoperative cardiac arrest
22	SIMON STRØYER [73]	2017	Retrospective Study	Ivor–Lewis Esophagectomy	234	SAS could not predict adverse outcomes	Not applicable	• Can’t predict

**Table 3:** All kind of surgeries

NO	Author(s)	Year	Type of study	Type of surgery	Number of patients	Article Findings	Surgical Apgar score	Main outcome
1	Paul Q. Reynolds [8]	2011	Cohort study	All kind of surgeries	123,864	There was a direct correlation between the SAS and the probability of Postoperative death	Not applicable	• Death • A weak association between SAS and mortality among burn patients
2	Kyoko Shiozaki [74]	2016	Prospectively study	craniotomy, neck surgery, thoracotomy, laparotomy, hip or pelvic surgery, spinal surge	284	SAS can predict serious unfavorable following major surgery	Not applicable	• Respiratory failure • Pulmonary embolism • Pulmonary edema • Acute myocardial infarction • Cardiac arrest • Deep venous thromboembolism • Acute kidney injury • Cerebrovascular disease • Severe sepsis • ICU readmission • Death
3	Maxim A. Terekhov [75]	2015	Retrospective cohort	Non cardiac surgery	44,835	This score alone was a considerable predictor of 30-day death	Not applicable	• 30 day mortality • Hospital mortality • Morbidity • ICU admission • Hospital readmission
4	Alex B. Haynes [76]	2011	Prospective observational study	Noncardiac Operation with general anesthesia	5,909	Predict major outcomes among adults who underwent non-cardiac surgery	SAS (0–4)	• site infection • unplanned return to the operating room • sepsis • prolonged mechanical ventilation • pneumonia
5	L. McLean House [77]	2016	Retrospective cohort study	Noncardiac surgery	46,799	SAS can improve patient triage	SAS (0–4)	• MI • sepsis • Cardiac troponin leak
6	Nina E. Glass [78]	2015	Prospective study	Any operation	2125	SAS is accompanied by a long ICU stay following elective postoperative admission	SAS ≤ 7	• Postoperative ICU Admission
7	Christopher C. Thorn [79]	2012	Prospective study	General surgical and vascular procedures, lower limb joint replacement, emergency fractured neck of the femur	223	SAS can predict emergency general and vascular outcomes It appears that the SAS is a useful adjunct in making decision in orthopedic surgery context	Not applicable	• acute renal failure • bleeding • cardiac arrest • deep venous thrombosis • myocardial infarction • unplanned intubation • pneumonia • pulmonary embolism • stroke • wound disruption • surgical site infection • sepsis • septic shock • systemic inflammatory response • syndrome vascular graft failure • death

**Table 4:** Main characteristics of included studies

NO	Author(s)	Year	Type of study	Type of surgery	Number of patients	Article Findings	Surgical Apgar score	Main outcome
1	Yoshitaka Aoki [80]	2018	Retrospective cohort study	Esophagectomy	246	eSAS maybe not be related to 30-day morbidity following esophagectomy. the modified eSAS was significantly related to major morbidity	modified eSAS < 4	• eSAS: Lack of relationship with major complications • modified eSAS can predict: pneumonia, sepsis, anastomotic stenosis, acute kidney injury, laryngeal nerve palsy, lymphorrhea,bronchial ulcer
2	Guan-Hua Li [81]	2021	Retrospective cohort study	Major abdominal surgery	1055	predictive for post-surgical delirium in those who undergo open abdominal procedures	SAS (0–2)	• Delirium
3	Maho Kinoshita [1]	2016	Prospective study	Surgery under general or regional anesthesia	24,318	The SAS is helpful for predicting 30-day mortality following surgery	not defined	• Death
4	Joseph A. Hyder [82]	2013	RCT	General or vascular surgery	3000	vital signs significantly impact the SAS	SAS (0–4)	• acute renal failure • bleeding • cardiac arrest • coma • deep venous thrombosis • myocardial infarction • unplanned intubation • ventilator use for 48 h or more • pulmonary embolism • stroke • wound disruption • Surgical site infection • Sepsis • Septic shock • Systemic inflammatory response syndrome • vascular graft failure
5	Yuichiro Miki [12]	2014	Retrospectiv study	Gastrectomy	328	modified SAS is considered a powerful predictor for developing intense outcomes in elective surgery	Modified SAS ≤ 6	• pancreatic fistula • anastomotic leakage • pleural effusion • bowel obstruction • abdominal abscess • bleeding • pneumonia • chylous ascites
6	Amy C. S. Pearson [83]	2017	Retrospective study	Liver transplantation	628	The Modified SAS predicted early morbidity and mortality after liver transplant	SAS (0–2)	• Sepsis • Reintubation New dialysis • Seizure • Myocardial infarction • Stroke • Pulmonary embolus • Death within 30 day • Postoperative cardiac arrest
7	Kristine E. Day [84]	2018	Retrospective study	Head and Neck Surgery	713	The mSAS demonstrates benefit in predicting patients who are in danger of post-surgical complications	Modified SAS (0–4)	• renal insufficiency • urinary tract infection • post-surgical blood transfusions
8	Seon Hee Park [85]	2018	Retrospective study	Robotic-assisted radical hysterectomy	138	MSAS is better in predicting intraoperative complications	Modified SAS ≤ 6	• Bleeding • Bowel injury • Bladder or ureter injury • Fever • Urinary distention • Ileus • Vault bleeding • Readmission • Lymphedema • dysrhythmia • Nerve palsy • Wound dehiscence • Fistula • Peritonitis
9	Guoping Ding [86]	2019	prospective cohort study	Pancreaticoduodenectomy (PD) and distal pancreatectomy(DP)	160	the modified SAS proposed in the present study, based on OT(operation time) instead of HR(heart rate), exert a better estimate value in pancreatic ductal adenocarcinoma patients	SAS < 8 Modified SAS < 7	• Pneumonia • pleural effusion • morbidity
10	Xue-Zhong Xing [87]	2016	retrospectively	open esophagectomy	189	e SAS is strongly correlated with hospitalization but not with admission to ICU or death	eSAS ≤ 7	• anastomotic leak • pulmonary infection combined with respiratory insufficiency • hospital length of stay
11	Yong Xi [88]	2020	Retrospective cohort	radical esophagectomy	194	This score can predict major outcomes	Not applicable	• Reintubation • Pneumonia • Anastomotic or conduit leak • ventilatory support > 48 h • Recurrent nerve paresis
12	Mehmet Nuri Yakar [89]	2021	retrospective observational study	emergency surgery	579	This score is utilized as a scoring model to predict post-operative mortality and complications	Not applicable	• impaired consciousness • The need for intensive care unit • The need for mechanical ventilation during • the first 48 h postoperatively • Unplanned intubation • Unplanned reoperation • Bleeding requiring transfusion • Postoperative blood and blood product • transfusion • Surgical site infection • Newly-emerging cardiac arrhythmia • Pneumonia • Bacteriemia • Acute renal failure • Cardiac arrest • Cardiopulmonary resuscitation • Albumin replacement • Inotropic support • 30day mortality
13	Christopher F. Janowak [90]	2015	Retrospective review	Esophagectomy	168	eSAS can independently predict 30—day major morbidity after esophagectomy	eSAS ≤ 6	• Reintubation • Prolonged ventilation • Pneumonia • Sepsis • Septic shock • Anastomotic leak • Chylothoraxz

Our research showed contradictory results regarding the use of SAS and modified SAS in different surgeries; for example, six studies represented that SAS was not an estimated factor for complications following surgery, but modified SAS was considered a valuable predictor for surgery complications (Tables 1, 2, 3, and 4).

In addition, a study found that the eSAS may not be correlated during 30-day significant malady following surgery. Nevertheless, the modified eSAS demonstrated a significant association with major morbidity. (Table 4).

From another point of view, in the review of 63 studies, it was revealed that the SAS could predict cardiovascular, respiratory, digestive, urogenital, neurological, systemic, and infectious [91] complications, the duration of hospitalization in the intensive care unit (ICU), mortality, and the survival rate in various surgeries (Tables 1, 2, 3, and 4). Additionally, two other studies demonstrated SAS weak differences for major and minor complications after surgeries (Table 1).

On the contrary, in 11 studies, it was found that SAS was not correlated with complications after surgeries (Tables 1, 2, 3, and 4). The obtained results are discussed in the following sections.

### General and vascular surgery

Regarding general and vascular surgery, there were a total of 14 studies in which SAS predicted complications after surgery (*n* = 12), death after surgery (*n* = 6), and the requirement to stay in the ICU (*n* = 2).

### Emergency surgery

In the field of emergency surgery, six studies were found in which SAS could predict complications after surgery (*n* = 5), died after surgery (*n* = 4), and the requirement to stay in ICU (*n* = 1), along with one non-prediction case.

### Thoracic surgery

Considering thoracic surgery, a total of 10 studies were obtained in which SAS predicted complications after surgery (*n* = 9), death after surgery (*n* = 1), and length of hospital stay (*n* = 2) in addition to one non-prediction case.

### Cancer surgery

Overall, 12 studies were found regarding cancer surgery, in which SAS could predict complications after surgery (*n* = 10), died after surgery (*n* = 3), and the requirement to stay in ICU (*n* = 1).

### Gynecological surgery

In the Gynecosurgery field, three studies were achieved, in which SAS predicted complications after surgery (*n* = 3), death after surgery (*n* = 1), and the requirement to stay in the ICU (*n* = 1).

### Liver and pancreas surgery

There were seven studies in emergency surgery [92] in which SAS could predict complications after surgery (*n* = 7) and death after surgery (*n* = 4).

### Orthopedic surgery

With regard to orthopedic surgery, there were a total of six studies in which SAS anticipated complications after surgery (*n* = 4), death after surgery (*n* = 1), and the requirement to stay in the ICU (*n* = 1), along with two non-prediction cases.

### Urological surgery

In the field of urological surgery, four studies were found in which SAS predicted complications after surgery (*n* = 2) and the requirement to stay in the ICU (*n* = 1), as well as one non-prediction case.

### Neurosurgery

Generally, six studies were related to neurosurgery, in which SAS could predict complications after surgery (*n* = 6) and death after surgery (*n* = 3).

### Head and neck surgery

Two studies were about head and neck surgery, in which SAS anticipated complications after surgery (*n* = 1), along with one non-prediction case.

### Other surgeries

As regards the other surgeries, eight studies were obtained in which SAS predicted complications after surgery (*n* = 4), died after surgery (*n* = 4), and the requirement to stay in ICU (*n* = 2) in addition to one non-prediction case.

Hence, after this study, it can be concluded that the "SAS,” “Postoperative complications," "Surgery," "Morbidity," “requirement to stay in ICU," and "Mortality" used SAS as a predictor instrument to identify the correlation with early and late postoperative outcomes. In addition, modifications in SAS (Modified SAS) or the combination of SAS with ASA criteria can help identify patients who require incessant monitoring and follow-up while going through the postoperative period.

## Discussion

Virginia Apgar initially developed the Apgar score in 1953 for assessing neonatal health and predicting morbidity and mortality shortly after birth. It was primarily designed for use in obstetrics and pediatrics to quickly evaluate the newborn's overall condition based on specific criteria such as heart rate, respiratory effort, muscle tone, reflex irritability, and color. The Apgar score has since become a widely used and standardized method for evaluating the immediate health status of newborns. Giugliano et al., by modifying and applying some changes in this score, designed the SAS in a way that it can predict complications and mortality during surgery [64]. The SAS is a straightforward scoring system from 0 to 10. it is derived from three during-procedure variables collected during surgery, which include LHR, EBL and MAP. Variables are used to Compute the SAS, concisely assessing a patient's physiological status during the surgical procedure [38]. Several studies have examined many data regarding SAS prospectively and retrospectively. The following section will discuss the results of these studies in detail.

The results obtained from these studies were categorized into four tables, including SAS results in general, vascular, oncological, and neurological (Table 1), as well as orthopedics, urology, gynecology/obstetrics, and thoracic (Table 2) surgeries, respectively. In addition, Tables 3 and 4 present SAS results in different surgeries and modified SAS, respectively. Many of these studies have demonstrated that SAS alone can be a valuable model for estimating complications after a variety of surgical specialties such as general [12, 32, 52], colorectal [17], gynecology [28], orthopedics [24], and neurosurgery [27] ones. Mastalerz et al. confirmed SAS < 8 for the prediction of thirty-day complications after surgery [93]. Likewise, Haynes et al. confirmed SAS globally in a multicenter clinical study in eight countries [76]. Some evidence indicated that SAS, combined with other criteria, has a better diagnostic ability to estimate complications after surgeries. Pinho et al. [50] examined Possum and SAS for their utility in determining whether to admit patients to the ICU right away following colorectal surgery; they found that Possum had greater sensitivity and specificity, but the drawback is that it needs a wide variety of clinical and laboratory data. In addition to the initial SAS, various iterations of the SAS were created by researchers to more accurately pinpoint the hazards related to particular patients or surgical groups.

In comparison to SAS and ASA used separately, evidence shows SASA (a compound of SAS and ASA) and came to the conclusion that this new version is more accurate at predicting postoperative problems [1]. In the investigation of Kotera et al., another combination of SAS was used in patients with femoral neck fracture; their results revealed that the combination of SAS with ASA class = 3 improves the capability to predict post-surgical complications [94]. In their study, Miki et al. analyzed the files of 328 people undergoing gastrectomy. They simultaneously used the original and modified SAS criteria to predict surgical results. It was found that mSAS (modified Surgical Apgar Score) was reported to be a valuable predictor for drastic outcomes succeeding gastrectomy. At the same time, oSAS (original Surgical Apgar Score) did not demonstrate the same predictive value [12]. Another version of SAS used in esophageal surgeries is called SAS. The results regarding eSAS are also contradictory. Janowak et al. reported that SAS ≤ 6 strongly predicts postoperative complications [90]. Xing et al.'s findings, indicated a powerful relation between the eSAS and the hospitalization period. However, they did not see an association between eSAS and the stay length in the ICU or the mortality rate [87]. Aoki et al. demonstrated that eSAS was not significantly related to major 30-day complications after esophagectomy [80]. The findings confirmed that eSAS is not an available universal score and seems to vary based on the type of the performed surgical technique; therefore, it needs re-evaluation.

From another point of view, the review of the conducted studies showed that the SAS has the ability to predict cardiovascular, respiratory, digestive, urogenital, neurological, systemic, and infectious complications, the duration of hospitalization in the ICU, mortality, and the survival rate. The SAS was found to be directly related to the development of pancreatic fistula following the operation in the paper by Asifi et al., which covered 553 people who underwent pancreaticoduodenectomy surgery. The 30-day mortality for these individuals was not significantly predicted by the SAS, though [36]. In addition, Reynolds et al., investigating the data of 123,864 surgical procedures that included all surgical specialties, concluded that the correlation between SAS and postoperative mortality on days 7, 30, and 90 varied based on the type of surgery [8]. This issue can be justified by considering the difference in co-morbidities and potential causes of death in each type of surgical specialty; for instance, SAS is more likely to predict major cardiac events in vascular patients than sepsis in burn patients. A different study conducted by Buzincu et al. noted that the SAS (Surgical Apgar Score) had limited ability to differentiate between patients who would experience complications following an operation and those who would not. However, despite this limitation, the SAS proved valuable in diagnosing patients at risk of prompt postoperative dysfunction of the organ.

Additionally, it was effective in predicting early postoperative cardiovascular complications that require inotrope/vasopressor therapy and metabolic disorders characterized by elevated serum lactate levels. These findings suggest that while the SAS may not excel in overall complication prediction, it can be valuable in identifying specific postoperative issues such as organ dysfunction, cardiovascular complications, and metabolic disturbances [37]. According to the study conducted by Glass et al. [78], an SAS value of less than eight was a valuable criterion for predicting the requirement of special care in those who underwent general procedures. In another survey involving 399 patients who underwent esophagectomy surgery, it was observed that the SAS had a significant correlation with the incidence of pulmonary complications, anastomosis leakage, and surgical site infection [63]. Finally, some evidence indicated that SAS was unable to predict outcomes in those undergoing knee arthroplasty [55], malignant hysterectomy [69], spine surgery for metastasis [11], gastrectomy [12], and cervical vascular reconstruction [95]. These studies discussed several reasons for the limited capability of the SAS to predict surgical outcomes. Some of the reasons are highlighted in the following paragraph.

Most of the studies were retrospective analyses in a single institution; therefore, there was a possibility for several biases. Hence, to evaluate the usefulness of this score, a prospective study with follow-ups on the potential effect of the score on the results is necessary. Furthermore, minor complications such as urinary tract infections may not be recorded in the discharge summary and electronic file. The evidence suggests that EBL has a high grade of mistake and varies depending on the performance of each center or person and the type of surgery; it can also increase the possibility of errors in the study results. Moreover, SAS is not extensively used in surgical specialties and may be considered more as an instrument to compare the research. Moreover, it is not known whether the proper control of these three variables (LHR, EBL & MAP) can improve patient outcomes. Considering the findings, it appears that the SAS should be changed in the future for improved prediction among each surgical subspecialty, even if it has already been validated in an expansive variety of surgical subspecialties.

## Conclusion

SAS is a straightforward system of scoring, which is easy to compute and record. SAS is independent of the kind of surgery (elective, urgent, or emergency) and does not require biochemical analyses, clinical assessments, or the classification of a disease as acute or chronic. Low SAS patients may experience difficulties following surgery for thirty days. Surgeons and anesthesiologists can recognize patients who are in danger thanks to the analysis of SAS. Furthermore, by modifying the SAS or combining it with ASA (American Society of Anesthesiologists) criteria, healthcare professionals can better identify patients who require to be continuously monitored and followed up in the postoperative period. This can help ensure timely interventions and appropriate care for patients with raised complications.

## Data Availability

This published article includes all data generated or analyzed during this study.

## Footnotes

1 - Modified SAS.

2 - Esophagectomy SAS.

3 -Modified esophagectomy SAS.

## References

[1] Kinoshita M, Morioka N, Yabuuchi M, Ozaki M (2017). New surgical scoring system to predict postoperative mortality. J Anesth.

[2] Singh K. Hariharan SJTJoA, Reanimation. Detecting Major Complications and Death After Emergency Abdominal Surgery Using the Surgical Apgar Score: A Retrospective Analysis in a Caribbean Setting. 2019;47(2):128. 10.5152/TJAR.2019.65872 PMC6499045 31080954

[3] Chhabra A, Singh A, Kuka PS, Kaur H, Kuka AS. Chahal HJNJoS. Role of perioperative surgical safety checklist in reducing morbidity and mortality among patients: An observational study. 2019;25(2):192–7. 10.4103/njs.NJS_45_18 PMC6771182 31579376

[4] Kinoshita M, Morioka N, Yabuuchi M. Ozaki MJJoa. New surgical scoring system to predict postoperative mortality. 2017;31(2):198–205. 10.1007/s00540-016-2290-2 PMC5378752 27995328

[5] Hariharan S. Zbar AJCs. Risk scoring in perioperative and surgical intensive care patients: a review. 2006;63(3):226–36. 10.1016/j.cursur.2006.02.005 16757378

[6] Sobol JB, Wunsch H. Triage of high-risk surgical patients for intensive care. Crit Care. 2011;15(2):217. 10.1186/cc9999. Epub 2011 Mar 22. 10.1186/cc9999 PMC3219413 21457500

[7] Zheng C, Luo C, Xie K, Li JS, Zhou H, Hu LW, Wang GM, Shen Y. Surgical Apgar score could predict complications after esophagectomy: a systematic review and meta-analysis. Interact Cardiovasc Thorac Surg. 2022;35(1):ivac045. 10.1093/icvts/ivac045. 10.1093/icvts/ivac045 PMC9714643 35293571

[8] Reynolds PQ, Sanders NW, Schildcrout JS, Mercaldo ND, St Jacques PJ. Expansion of the surgical Apgar score across all surgical subspecialties as a means to predict postoperative mortality. Anesthesiology. 2011;114(6):1305–12. 10.1097/ALN.0b013e318219d734. 10.1097/ALN.0b013e318219d734 21502856

[9] Aoki Y, Ide K, Nakajima F, Kawasaki Y, Fujita Y, Morimoto E, et al., editors. Esophagectomy surgical Apgar score may not be associated with postoperative morbidity. Seminars in Thoracic and Cardiovascular Surgery; 2019: Elsevier. 10.1053/j.semtcvs.2018.12.002 30529159

[10] Grigorescu BL, Săplăcan I, Petrișor M, Bordea IR, Fodor R, Lazăr A. Perioperative Risk Stratification: A Need for an Improved Assessment in Surgery and Anesthesia-A Pilot Study. Medicina (Kaunas). 2021;57(10):1132. 10.3390/medicina57101132. 10.3390/medicina57101132 PMC8538842 34684169

[11] Lau D, Yee TJ, La Marca F, Patel R, Park PJCSS. Utility of the Surgical Apgar Score for patients who undergo surgery for spinal metastasis. 2017;30(8):374–81. 10.1097/BSD.0000000000000174 28937460

[12] Miki Y, Tokunaga M, Tanizawa Y, Bando E, Kawamura T, Terashima M (2014). Preoperative risk assessment for gastrectomy by surgical apgar score. Ann Surg Oncol..

[13] Urrutia J, Valdes M, Zamora T, Canessa V, Briceno JJIo. Can the Surgical Apgar Score predict morbidity and mortality in general orthopaedic surgery? 2012;36(12):2571–6. 10.1007/s00264-012-1696-1 PMC3508032 23129225

[14] Pittman E, Dixon E, Duttchen K (2022). The Surgical Apgar Score: A Systematic Review of Its Discriminatory Performance. Annals of Surgery Open.

[15] Peters JP, Hooft L, Grolman W, Stegeman I (2015). Reporting quality of systematic reviews and meta-analyses of otorhinolaryngologic articles based on the PRISMA statement. PLoS ONE.

[16] Scale NO. Wells GA, Shea B, O’Connell D, Peterson J, Welch V, Losos M, et al. The Newcastle-Ottawa Scale (NOS) for assessing the quality of nonrandomised studies in meta-analyses.

[17] Regenbogen SE, Bordeianou L, Hutter MM, Gawande AA (2010). The intraoperative Surgical Apgar Score predicts postdischarge complications after colon and rectal resection. Surgery.

[18] Regenbogen SE, Lancaster RT, Lipsitz SR, Greenberg CC, Hutter MM, Gawande AA (2008). Does the Surgical Apgar Score measure intraoperative performance?. Ann Surg.

[19] Singh K, Hariharan S (2019). Detecting Major Complications and Death After Emergency Abdominal Surgery Using the Surgical Apgar Score: A Retrospective Analysis in a Caribbean Setting. Turkish journal of anaesthesiology and reanimation.

[20] Sobol JB, Gershengorn HB, Wunsch H, Li G (2013). The surgical Apgar score is strongly associated with intensive care unit admission after high-risk intraabdominal surgery. Anesth Analg.

[21] Sugimoto A, Fukuoka T, Nagahara H, Shibutani M, Iseki Y, Sasaki M (2022). The impact of the surgical Apgar score on oncological outcomes in patients with colorectal cancer: a propensity score-matched study. World J Surg Oncol.

[22] Tomimaru Y, Takada K, Shirakawa T, Noguchi K, Morita S, Imamura H (2018). Surgical Apgar score for predicting complications after hepatectomy for hepatocellular carcinoma. J Surg Res.

[23] Toyonaga Y, Asayama K, Maehara Y (2017). Impact of systemic inflammatory response syndrome and surgical Apgar score on post-operative acute kidney injury. Acta Anaesthesiol Scand.

[24] Urrutia J, Valdes M, Zamora T, Canessa V, Briceno J (2015). An assessment of the Surgical Apgar Score in spine surgery. The spine journal : official journal of the North American Spine Society.

[25] Yamada T, Tsuburaya A, Hayashi T, Aoyama T, Fujikawa H, Shirai J, et al. Usefulness of Surgical Apgar Score on Predicting Survival After Surgery for Gastric Cancer. Ann Surg Oncol. 2016;23:S757–63. 10.1245/s10434-016-5525-4 27557829

[26] Yu W, Huang C, Wang Q, Zhao E, Ding Y, Huang T (2016). Plasma BNP level combined with surgical Apgar score to predict operative major cardiac adverse events in malignant obstructive jaundice patients. Pakistan Journal of Medical Sciences.

[27] Ziewacz JE, Davis MC, Lau D, El-Sayed AM, Regenbogen SE, Sullivan SE (2013). Validation of the surgical Apgar score in a neurosurgical patient population. J Neurosurg.

[28] Zighelboim I, Kizer N, Taylor NP, Case AS, Gao F, Thaker PH (2010). "Surgical Apgar Score" predicts postoperative complications after cytoreduction for advanced ovarian cancer. Gynecol Oncol.

[29] Jering MZ, Marolen KN, Shotwell MS, Denton JN, Sandberg WS, Ehrenfeld JM (2015). Combining the ASA Physical Classification System and Continuous Intraoperative Surgical Apgar Score Measurement in Predicting Postoperative Risk. J Med Syst.

[30] Kenig J, Mastalerz K, Lukasiewicz K, Mitus-Kenig M, Skorus U (2018). The Surgical Apgar Score predicts outcomes of emergency abdominal surgeries both in fit and frail older patients. Arch Gerontol Geriatr..

[31] Kenig J, Mastalerz K, Mitus J, Kapelanczyk A (2018). The Surgical Apgar score combined with Comprehensive Geriatric Assessment improves short- but not long-term outcome prediction in older patients undergoing abdominal cancer surgery. J Geriatr Oncol.

[32] La Torre M, Ramacciato G, Nigri G, Balducci G, Cavallini M, Rossi M (2013). Post-operative morbidity and mortality in pancreatic surgery. The role of surgical Apgar score. Pancreatology.

[33] Masi A, Amodeo S, Hatzaras I, Pinna A, Rosman AS, Cohen S (2017). Use of the surgical Apgar score to enhance Veterans Affairs Surgical Quality Improvement Program surgical risk assessment in veterans undergoing major intra-abdominal surgery. Am J Surg.

[34] Aoyama T, Katayama Y, Murakawa M, Yamaoku K, Kanazawa A, Higuchi A (2016). Risk assessment of pancreatic surgery by surgical apgar score and body mass index. Int Surg.

[35] Arifin MZ, Sendjaja AN, Faried A (2021). Application of the surgical apgar score (Sas) to predict postoperative complication(s) in the patients with traumatic brain injury: Study of single center in Indonesia. Open Access Macedonian Journal of Medical Sciences.

[36] Assifi MM, Lindenmeyer J, Leiby BE, Grunwald Z, Rosato EL, Kennedy EP (2012). Surgical Apgar score predicts perioperative morbidity in patients undergoing pancreaticoduodenectomy at a high-volume center. J Gastrointest Surg.

[37] Buzincu I, Tănase S, Puf C, Ristescu I, Rusu DM, Pătrășcanu E (2021). Surgical Apgar Score predictive value for early postoperative organ dysfunction in cancer patients. Acta Chir Belg..

[38] Cihoric M, Toft Tengberg L, Bay-Nielsen M, Bang FN (2016). Prediction of Outcome After Emergency High-Risk Intra-abdominal Surgery Using the Surgical Apgar Score. Anesth Analg.

[39] Ettinger KS, Moore EJ, Lohse CM, Reiland MD, Yetzer JG, Arce K (2016). Application of the Surgical Apgar Score to Microvascular Head and Neck Reconstruction. J Oral Maxillofac Surg.

[40] Goel N, Manstein SM, Ward WH, DeMora L, Smaldone MC, Farma JM (2018). Does the Surgical Apgar Score predict serious complications after elective major cancer surgery?. J Surg Res.

[41] Gothwal S, Mohan A, Khan F, Om P (2019). Comparison of Major Complication Rate in High and Low Surgical Apgar Score in Abdominal Surgery Cases. Indian Journal of Surgery.

[42] Hsu SY, Ou CY, Ho YN, Huang YH (2017). Application of Surgical Apgar Score in intracranial meningioma surgery. PLoS ONE.

[43] Mitsiev I, Rubio K, Ranvir VP, Yu D, Palanisamy AP, Chavin KD (2021). Combining ALT/AST Values with Surgical APGAR Score Improves Prediction of Major Complications after Hepatectomy. Journal of surgery and research.

[44] Miura K, Koda M, Funayama T, Takahashi H, Noguchi H, Mataki K (2022). Surgical Apgar Score and Controlling Nutritional Status Score are significant predictors of major complications after cervical spine surgery. Sci Rep.

[45] Muthuvel G, Tevis SE, Liepert AE, Agarwal SK, Kennedy GD (2014). A composite index for predicting readmission following emergency general surgery. J Trauma Acute Care Surg.

[46] Ngarambe C, Smart BJ, Nagarajan N, Rickard J (2017). Validation of the Surgical Apgar Score After Laparotomy at a Tertiary Referral Hospital in Rwanda. World J Surg.

[47] Ohlsson H, Winso O (2011). Assessment of the Surgical Apgar Score in a Swedish setting. Acta Anaesthesiol Scand.

[48] Ou CY, Hsu SY, Huang JH, Huang YH (2017). Surgical apgar score in patients undergoing lumbar fusion for degenerative spine diseases. Clin Neurol Neurosurg.

[49] Padilla-Leal KE, Flores-Guerrero JE, Medina-Franco H (2021). Surgical Apgar score as a complication predictor in gastrointestinal oncologic surgery. Rev Gastroenterol Mex (Engl Ed).

[50] Pinho S, Lagarto F, Gomes B, Costa L, Nunes CS, Oliveira C (2018). CR-POSSUM and Surgical Apgar Score as predictive factors for patients’ allocation after colorectal surgery. Brazilian Journal of Anesthesiology.

[51] Gawande AA, Kwaan MR, Regenbogen SE, Lipsitz SA (2007). Zinner MJJJotACoS. An Apgar score for surgery.

[52] Melis M, Pinna A, Okochi S, Masi A, Rosman AS, Neihaus D (2014). Validation of the Surgical Apgar Score in a veteran population undergoing general surgery.

[53] Sakan S, Pavlovic DB, Milosevic M, Virag I, Martinovic P, Dobric I (2015). Implementing the Surgical Apgar Score in patients with trauma hip fracture. Injury.

[54] Wied C, Foss NB, Kristensen MT, Holm G, Kallemose T, Troelsen A (2016). Surgical apgar score predicts early complication in transfemoral amputees: Retrospective study of 170 major amputations. World journal of orthopedics.

[55] Wuerz TH, Regenbogen SE, Ehrenfeld JM, Malchau H, Rubash HE, Gawande AA (2011). The Surgical Apgar Score in Hip and Knee Arthroplasty. Clin Orthop Rel Res.

[56] Stoll WD, Taber DJ, Palesch SJ, Hebbar L (2016). Utility of the surgical apgar score in kidney transplantation: Is it feasible to predict ICU admission, hospital readmission, length of stay, and cost in this patient population?. Prog Transplant.

[57] Kotera A (2018). The Surgical Apgar Score can help predict postoperative complications in femoral neck fracture patients: a 6-year retrospective cohort study. Ja Clinical Reports.

[58] Ito T, Abbosh PH, Mehrazin R, Tomaszewski JJ, Li T, Ginzburg S (2015). Surgical Apgar Score predicts an increased risk of major complications and death after renal mass excision.

[59] Orberger M, Palisaar J, Roghmann F, Mittelstadt L, Bischoff P, Noldus J (2017). Association between the Surgical Apgar Score and Perioperative Complications after Radical Prostatectomy. Urol Int.

[60] Haroon F, Younus S, Peracha A, Memon N, Memon N (2021). Predictability of Surgical Apgar Score for postoperative outcomes in hip fractures: A prospective observational study. Journal of Acute Disease.

[61] Hayashi M, Kawakubo H, Mayanagi S, Nakamura R, Suda K, Wada N (2019). A low surgical Apgar score is a predictor of anastomotic leakage after transthoracic esophagectomy, but not a prognostic factor. Esophagus.

[62] Nagoya A, Kanzaki R, Kimura K, Fukui E, Kanou T, Ose N, et al. Utility of the surgical Apgar score for predicting the short- and long-term outcomes in non-small-cell lung cancer patients who undergo surgery. Interactive Cardiovascular and Thoracic Surgery.11. 10.1093/icvts/ivac150 PMC9297508 35640534

[63] Eto K, Yoshida N, Iwatsuki M, Kurashige J, Ida S, Ishimoto T (2016). Surgical Apgar Score Predicted Postoperative Morbidity After Esophagectomy for Esophageal Cancer. World J Surg.

[64] Giugliano DN, Morgan A, Palazzo F, Leiby BE, Evans NR, Rosato EL (2017). Surgical Apgar score (SAS) predicts perioperative morbidity, mortality, and length of stay in patients undergoing esophagectomy at a high-volume center. J Surg Oncol.

[65] Yamamoto M, Kurata K, Asai-Sato M, Shiomi M, Ueda Y, Aoki Y (2021). Low surgical Apgar score in older patients with gynecological cancer is a risk factor for postoperative complications and 1-year mortality: A multicenter retrospective cohort study. Mol Clin Oncol.

[66] Kurata K, Chino Y, Shinagawa A, Kurokawa T, Yoshida Y (2017). Surgical Apgar Score predicts 30-day morbidity in elderly patients who undergo non-laparoscopic gynecologic surgery: A retrospective analysis. Int J Surg.

[67] Bhandoria G, Mane JD (2020). Can Surgical Apgar Score (SAS) Predict Postoperative Complications in Patients Undergoing Gynecologic Oncological Surgery?. Indian J Surg Oncol.

[68] Bhandoria G, Mankad M, Dave P, Desai A, Patel S. Surgical Apgar Score: Validation in a Regional Cancer Centre in Western India. Indian Journal of Gynecologic Oncology. 2017;15(3).

[69] Clark RM, Lee MS, Alejandro Rauh-Hain J, Hall T, Boruta DM, del Carmen MG (2015). Surgical Apgar Score and prediction of morbidity in women undergoing hysterectomy for malignancy. Gynecol Oncol.

[70] Nakagawa A, Nakamura T, Oshikiri T, Hasegawa H, Yamamoto M, Kanaji S (2017). The Surgical Apgar Score Predicts Not Only Short-Term Complications But Also Long-Term Prognosis After Esophagectomy. Ann Surg Oncol.

[71] Prasad SM, Ferreria M, Berry AM, Lipsitz SR, Richie JP, Gawande AA (2009). Surgical apgar outcome score: perioperative risk assessment for radical cystectomy.

[72] Amy C. S. Pearson M, 1 Arun Subramanian, MBBS,2 Darrell R. Schroeder, MS,3 and James Y. Findlay, MB, ChB2. Adapting the Surgical Apgar Score for Perioperative Outcome Prediction in Liver Transplantation: A Retrospective Study. 2017. 10.1097/TXD.0000000000000739 PMC5682766 29184910

[73] Strøyer S, Mantoni T (2017). Svendsen LBJJoSO. Evaluation of the surgical Apgar score in patients undergoing Ivor-Lewis esophagectomy.

[74] Shiozaki K, Morimatsu H, Matsusaki T, Iwasaki A (2016). Observational Study to Assess and Predict Serious Adverse Events after Major Surgery. Acta Med Okayama.

[75] Terekhov MA, Ehrenfeld JM, Wanderer JP (2015). Preoperative Surgical Risk Predictions Are Not Meaningfully Improved by Including the Surgical Apgar Score: An Analysis of the Risk Quantification Index and Present-On-Admission Risk Models. Anesthesiology.

[76] Haynes AB, Regenbogen SE, Weiser TG, Lipsitz SR, Dziekan G, Berry WR (2011). Surgical outcome measurement for a global patient population: validation of the Surgical Apgar Score in 8 countries. Surgery.

[77] House LM, Marolen KN, St Jacques PJ, McEvoy MD, Ehrenfeld JM (2016). Surgical Apgar score is associated with myocardial injury after noncardiac surgery. J Clin Anesth.

[78] Glass NE, Pinna A, Masi A, Rosman AS, Neihaus D, Okochi S (2015). The surgical apgar score predicts postoperative ICU admission.

[79] Thorn CC, Chan M, Sinha N (2012). Harrison RAJWjos. Utility of the Surgical Apgar Score in a district general hospital.

[80] Aoki Y, Ide K, Nakajima F, Kawasaki Y, Fujita Y, Morimoto E (2019). Esophagectomy Surgical Apgar Score May Not Be Associated With Postoperative Morbidity. Semin Thorac Cardiovasc Surg.

[81] Li GH, Zhao L, Lu Y, Wang W, Ma T, Zhang YX (2021). Development and validation of a risk score for predicting postoperative delirium after major abdominal surgery by incorporating preoperative risk factors and surgical Apgar score. J Clin Anesth.

[82] Hyder JA, Kor DJ, Cima RR, Subramanian A (2013). How to improve the performance of intraoperative risk models: an example with vital signs using the surgical apgar score. Anesth Analg.

[83] Pearson AC, Subramanian A, Schroeder DR, Findlay JYJTd. Adapting the surgical Apgar score for perioperative outcome prediction in liver transplantation: a retrospective study. 2017;3(11). 10.1097/TXD.0000000000000739 PMC5682766 29184910

[84] Day KE, Prince AC, Lin CP, Greene BJ (2018). Carroll WRJOH, Surgery N. Utility of the modified surgical Apgar score in a head and neck Cancer population.

[85] Park SH, Lee J-Y, Nam EJ, Kim S, Kim SW (2018). Kim YTJBc. Prediction of perioperative complications after robotic-assisted radical hysterectomy for cervical cancer using the modified surgical Apgar score.

[86] Ding G, Zhou L, Chen W, Wu Z, Shen T, Cao LJL,Endoscopic,  (2019). Utility of the Surgical Apgar Score in pancreatic cancer and modification.

[87] Xing XZ, Wang HJ, Qu SN, Huang CL, Zhang H, Wang H, et al. The value of esophagectomy surgical apgar score (eSAS) in predicting the risk of major morbidity after open esophagectomy. J Thorac Dis. 2016;8(7):1780–7. 10.21037/jtd.2016.06.28 PMC4958794 27499969

[88] Xi Y, Shen W, Wang L, Yu C (2020). An esophagectomy Surgical Apgar Score (eSAS)-based nomogram for predicting major morbidity in patients with esophageal carcinoma. Translational cancer research.

[89] Yakar MN, Polat C, Akkılıç M, Yeşildal K, Duran Yakar N, Turgut N. Use of a modified surgical APGAR score for prediction of postoperative complications in emergency surgery: An observational retrospective study. Ulusal travma ve acil cerrahi dergisi = Turkish journal of trauma & emergency surgery : TJTES. 2022;28(5):615–25. 10.14744/tjtes.2021.34732 PMC10442991 35485468

[90] Janowak CF, Blasberg JD, Taylor L, Maloney JD (2015). Macke RAJTJot, surgery c. The surgical Apgar score in esophagectomy.

[91] Khah AMM, Khoozani AB (2020). How to protect operating room staff from COVID-19?. Perioperative care and operating room management.

[92] Merajikhah A, Beigi-Khoozani A, Soleimani M (2021). Risk of spreading delta coronavirus to operating room personnel after COVID-19 vaccination. Disaster and Emergency Medicine Journal.

[93] Mastalerz K, Kenig J, Olszewska U, Michalik CJV, Techniques OM. The Surgical Apgar score and frailty as outcome predictors in short-and long-term evaluation of fit and frail older patients undergoing elective laparoscopic cholecystectomy–a prospective cohort study. 2018;13(3):350–7. 10.5114/wiitm.2018.75878 PMC6174164 30302148

[94] Kotera A (2018). The Surgical Apgar Score can help predict postoperative complications in femoral neck fracture patients: a 6-year retrospective cohort study. Ja Clin Rep.

[95] Ettinger KS, Moore EJ, Lohse CM, Reiland MD, Yetzer JG, Arce K. Application of the Surgical Apgar Score to Microvascular Head and Neck Reconstruction. Journal of oral and maxillofacial surgery : official journal of the American Association of Oral and Maxillofacial Surgeons. 2016;74(8):1668–77. 10.1016/j.joms.2016.02.013 26997211

